# How is the weather? Forecasting inpatient glycemic control

**DOI:** 10.4155/fsoa-2017-0066

**Published:** 2017-09-11

**Authors:** George E Saulnier, Janna C Castro, Curtiss B Cook, Bithika M Thompson

**Affiliations:** 1 Department of Information Technology, Mayo Clinic Hospital, Phoenix, AZ 85054, USA; 2 Division of Endocrinology, Mayo Clinic Hospital, Phoenix, AZ 85054, USA; 3 Division of Preventive, Occupational & Aerospace Medicine, Mayo Clinic, Scottsdale, AZ 85259, USA

**Keywords:** forecasting, hyperglycemia, inpatient, operational research

## Abstract

**Aim:**

Apply methods of damped trend analysis to forecast inpatient glycemic control.

**Method:**

Observed and calculated point-of-care blood glucose data trends were determined over 62 weeks. Mean absolute percent error was used to calculate differences between observed and forecasted values. Comparisons were drawn between model results and linear regression forecasting.

**Results:**

The forecasted mean glucose trends observed during the first 24 and 48 weeks of projections compared favorably to the results provided by linear regression forecasting. However, in some scenarios, the damped trend method changed inferences compared with linear regression. In all scenarios, mean absolute percent error values remained below the 10% accepted by demand industries.

**Conclusion:**

Results indicate that forecasting methods historically applied within demand industries can project future inpatient glycemic control. Additional study is needed to determine if forecasting is useful in the analyses of other glucometric parameters and, if so, how to apply the techniques to quality improvement.

## Lay abstract

Hyperglycemia (high blood sugars) in the hospital can lead to complications, such as more surgical infections or longer hospital stays. Therefore, it is important to project how hyperglycemia may change over time to develop plans to intervene early. This study uses a mathematical technique used in industry called damped trend exponential smoothing analysis that allows forecasting of blood sugars into the future. At a population level, the method could allow hospitals to predict if hyperglycemia will get worse so that plans could be developed early to proactively prevent the problem from occurring.

The ability to analyze and report glucose data, so-called glucometrics, is essential to any quality improvement initiative associated with the care of hospitalized patients with diabetes mellitus [1]. Although measuring inpatient glucose data is assumed to lead to better outcomes, no data exist indicating that hospitals that do so have better outcomes than those that do not. Nonetheless, a number of glucometric gauges have been proposed [2,3]. The drawback of these current approaches is that the analyses rely on retrospective data. Statistical process control, or control charting, has been suggested as one method of ascertaining whether glycemic control remains in predefined limits, but even this approach uses retrospective data and would not identify an out-of-control process until after it had occurred [4,5]. At a population health level, the ability to forecast inpatient glycemic control could provide an opportunity to anticipate unfavorable changes at an institutional level before they become a problem. Diving deeper into subpopulations, interventions targeting specific inpatient locations might improve the care of patients on a day-to-day basis.

One approach to forecasting inpatient glucose data is to apply mathematical models employed in operational research. Models that forecast trends derived from time series data have been well established in commerce and include such applications as supply chain management, diffusion of new products, credit risk and accounting and finance [6–10].

To satisfy currently recommended inpatient glucose target ranges, inpatient practitioners must tightly manage glucose levels while trying to avoid wide swings in values. An appropriate forecasting model, the authors contend, would therefore accommodate the tight management of glucose levels by accounting for the natural attenuation of trends over time. While another mathematical technique used to project trends, linear regression, is simple to implement using common desktop tools, it may fail to provide the flexibility to account for natural attenuations [11,12]. Due to the damped trend forecasting method's ability to decrease the significance of trends when the behavior of underlying data dictates, it is hypothesized that this method improves upon the results of linear regression forecasting.

Evidence of an increasing or decreasing forecast in a patient population may represent a number of possible situations worthy of investigation or intervention. The effect of the intervention would be to stabilize the patient population and to soften the trend over time. The purpose of this analysis was to evaluate the utility of using a damped forecasting model as a predictive method of assessing population-based inpatient glycemic control compared with a linear regression forecasting technique.

## Methods

### Description of damped trend model

The damped trend exponential smoothing mathematical model applied in this analysis is well established and was developed to improve forecasting of inventory systems [6,7]. Before the damped trend model was used, inventory systems provided forecasts that were generally either too optimistic or too pessimistic [7]. This led to overstocking of goods when the optimistic demand for a particular product softened or product shortfalls when the decline of demand was overstated. A need was identified to establish a method that lessened the impact of a trend as the forecast extended farther into the future.

The damped trend exponential smoothing method uses three parameters to define the behavior of the model [7,8]:

- Alpha, *α*, the smoothing constant, decreases the variability of the model in relation to the observed values. Alpha is a component of the level function and can vary in value from 0 to 1.
- Gamma, *γ*, the trending constant, decreases the influence of trends in the model in relation to any trending visible in the observed values. Gamma is a component of the growth rate function and can vary in value between 0 and 1.
- Phi, *φ*, the damping constant, is used in both the level and growth rate functions and works to influence how quickly the growth rate of the previous period is dampened. The damping constant is a discounting factor used to curtail the propagation of a trend. A larger *φ* enables a trend to propagate longer than a smaller one. The damping constant can vary in value between 0 and 1.

The method applies two interdependent linear functions to model a time series in a manner that decreases the importance of an underlying trend as time progresses.

- The level function states that the estimate of the level made in the current time period, *l_T_*, consists of a fraction, alpha (α), of the observed value, *y_T_*, plus a complementary fraction (1–α) of the estimate, *l_T_*_−1_, and damped growth rate, *φb_T_*_−1_, made 1 period previous. The smaller the *α*, the less the model is influenced by the most recent observation and the greater the smoothing.
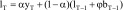

- The growth rate function states that the estimate of the growth rate made in the current time period, *b_T_*, consists of a fraction, gamma [γ], of the difference between the current and previous level estimates, *l_T_* − *l_T_*_−1_, plus a complementary fraction [1 – γ] of the damped growth rate, *φb_T_*_−1_, made 1 period previous. The larger the *γ*, the more the model is influenced by the most recent change in the level function.
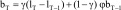

Fitting the damped trend exponential smoothing model to the observed data requires simultaneously solving the interdependent level and growth rate functions such that the absolute error between the model and the observations is minimized.

Once the optimal *α, γ* and *φ* parameters are identified, a point forecast, 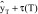, from time *T* to a future time period *τ* can be calculated with
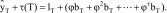



In the forecast, the variables *l_T_* and *b_T_* are the last known values of the level and growth rate functions. At each successive interval in the forecast, the last known level is augmented by an increasingly attenuated growth rate. The attenuation, or damping, is evident in the increasing exponent to *φ*. Because *φ* is a positive value less than 1, the exponent serves to dampen the growth rate the further the prediction is extended.

Finally, a 95% prediction interval forecast [8] may be computed using
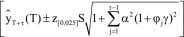



where φ_j_ = φ+φ^2^+…+φ^j^.

Linear regression forecasting models, by contrast, use the observed values to define parameters of the function:
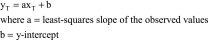



For a given set of observations, the slope and y-intercept are easily calculated using Microsoft Excel or similar programs. Forecasting beyond the observed values involves extending the straight line, *y_T_*, to the desired interval [11,12].

### Glucose data

Point-of-care glucose (POC-BG) data were extracted from the institution's laboratory information system linked to the electronic medical record as previously described [4,5]. Data were extracted from 2010 to 2014. A total of 55,328 patient-day-weighted mean glucose values were calculated from the POC-BG data. The patient-day-weighted mean reflects an aggregate glucose measure of a specific patient population – in this case, inpatients with diabetes mellitus who were not in an intensive care unit (ICU). This analysis did not involve any patient identifiers and was part of overall quality improvement/quality assurance effort on inpatient glycemic control, and Institutional Review Board determined that formal review and approval were not needed.

### Analysis

To ensure sufficient data were available to establish an adequate forecast, between 60 and 63 weekly observations were gathered for each scenario [10], timed to ensure that the forecast would begin at the start of a quarter. Then, short- and long-range forecast intervals were derived that extended 2, 4, 6, 24 and 48 weeks into the future. This resulted in the assessment of 100 different quarter/forecast-interval combinations (20 quarters × 5 forecast-intervals). The mean absolute percent error (MAPE) was the metric by which the accuracy of both the damped trend and linear regression forecasts were measured.

In demand-based industries, models resulting in MAPE values in the range of 10–15% appeared to be reasonable representations of the underlying data [10]. Performance of damped trend versus linear regression was compared for all 20 quarters analyzed, and examples were chosen that best illustrated the methodology. Four results of the analyses are provided and discussed as examples of the inpatient glucose forecasting methodology.

## Results

Of the 100 quarter/forecast-interval combinations, the damped trend method resulted in smaller MAPE values than the equivalent linear regression 65% of the time (Table 1). At every forecast interval, the damped trend method outperformed the linear regression at ratios from 1.5:1 (6-week and 24-week forecasts) to 2.3:1 (2-week and 48-week forecasts). Invariably, when the model outperformed the linear regression, the gap between the MAPE values increased as the forecast intervals increased. This suggests that, when compared with linear regression forecasts, the effect of attenuation enables the damped trend method to better represent the underlying data as the forecast interval extends further into the future.

**Table 1.** **Comparison of quarters from 2010 to 2014 where damped trend forecasting outperformed linear regression.**

**Forecasting method**	**Time period**					

	***2-week***	***4-week***	***6-week***	***24-week***	***48-week***	***Total***
Damped trend	14	13	12	12	14	65

Linear regression	6	7	8	8	6	35

Total quarters	20	20	20	20	20	100

### Example 1

**Figure 1.**
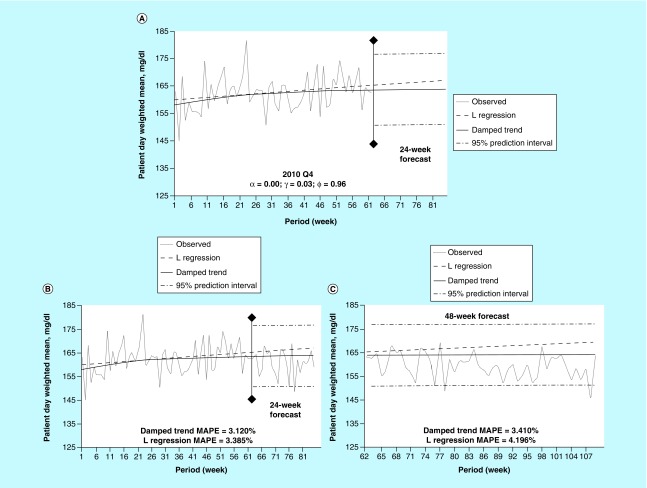
**Damped trend analysis for 2010.** **(A)** Damped trend and linear regression forecasts into the first 24 weeks without observed data for the time period. **(B)** The same comparisons as A with overlaying of observed values. **(C)** Damped trend and linear regression forecasts into the next 48 weeks of data. Each observation is the aggregation of a week-long period. MAPE: Mean absolute percent error; Q: Quarter.

Figure 1 illustrates the results of a forecasting analysis for POC-BG data derived from observations from 2010. Shown in Figure 1A are the observed (actual) preforecasted patient-day-weighted mean glucose values, a linear regression forecast, the damped trend prediction, damped trend prediction intervals and the optimal *α, γ* and *φ* parameters used (also shown in subsequent examples) derived from fourth quarter data. During the first 24 forecasted weeks (approximately weeks 62–85), the trajectories of the linear regression and damped trend lines diverge as the forecast interval progresses. When overlaying the observed values during the first 24 forecasted weeks (Figure 1B), the MAPE between the damped forecast and observed values was 3.120%, which compares favorably to the linear regression error of 3.385%. Beyond the first 24 forecasted weeks (Figure 1C), the forecasted and observed trend lines continued to track well compared with the linear regression, with a lower observed MAPE (3.410 vs 4.196%) after 48 weeks.

However because the model returned a damping coefficient *φ* of 0.96, the linear regression line diverges upward in relation to the damped forecast line, which remains flat through week 48. Had the prediction relied on a linear regression alone, the inference would have been that glucose values were increasing and that some intervention might be necessary to change glucose control processes when, it would appear, that would not have been needed. Such inappropriate intensification to lower glucose could result in increased risk of hypoglycemia.

### Example 2

**Figure 2.**
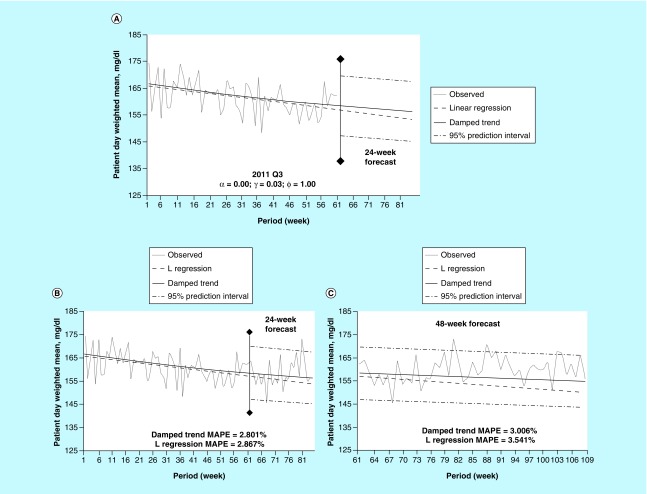
**Damped trend analysis for 2011.** **(A)** Damped trend and linear regression forecasts into the first 24 weeks without observed data for the time period. **(B)** The same comparisons as A with overlaying of observed values. **(C)** Damped trend and linear regression forecasts into the next 48 weeks of data. Each observation is the aggregation of a week-long period. MAPE: Mean absolute percent error; Q: Quarter.

Figure 2 illustrates data from third quarter 2011, with data forecasted initially into the first 24 weeks (Figure 2A), as in Figure 1. Observed and trended data are once again shown. The damped trend MAPE of 2.801% (Figure 2B) indicates a better agreement between the model and actual POC-BG control than an equivalent linear regression (MAPE = 2.867%). The gap between the model and the linear regression continues to grow through the 48-week time interval, with the model depicting a shallower rate of descent (Figure 2C) and a smaller MAPE (3.006 vs 3.541%). The downward slope of the linear regression line away from the dampedtrend line might infer that glucose control process were too tight. This could result in inappropriate loosening of glucose control processes when such interventions would not be needed. The long term impact of such a decision would be more hyperglycemia.

### Example 3

**Figure 3.**
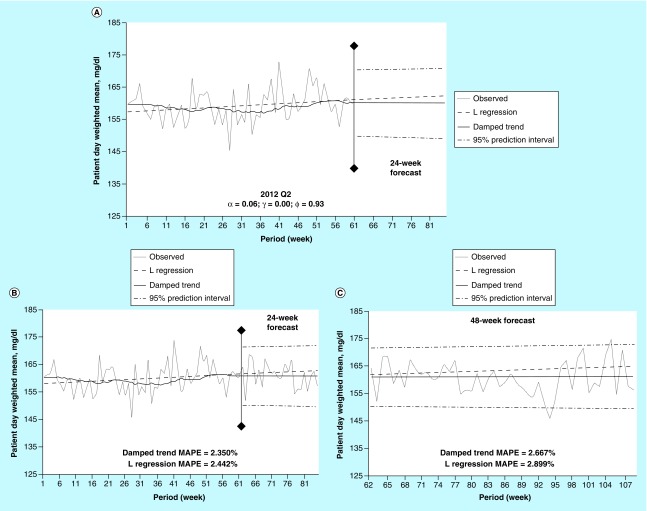
**Damped trend analysis for 2012.** **(A)** Damped trend and linear regression forecasts into the first 24 weeks without observed data for the time period. **(B)** The same comparisons as A with overlaying of observed values. **(C)** Damped trend and linear regression forecasts into the next 48 weeks of data. Each observation is the aggregation of a week-long period. MAPE: Mean absolute percent error; Q: Quarter.

In this third example (Figure 3), trending data for patient-day-weighted mean POC-BG levels are shown for 2012, with projections made for the second quarter of the year (Figure 3A). As with the previous illustrations, favorable agreement is evident (Figure 3B) between the model and observed glucose values (MAPE = 2.350%) for the first forecasted 24 weeks. Beyond the first 24 weeks (Figure 3C), divergence once again was seen between the linear regression and model trend lines, with the model exhibiting smaller error (MAPE = 2.667 vs 2.899%). As in the first example, a damping coefficient *φ* of 0.93 results in a flat trend line for the model where the linear regression depicts an increasing trend. Relying on a linear regression alone might result in a misinformed need for corrective action.

### Example 4

**Figure 4.**
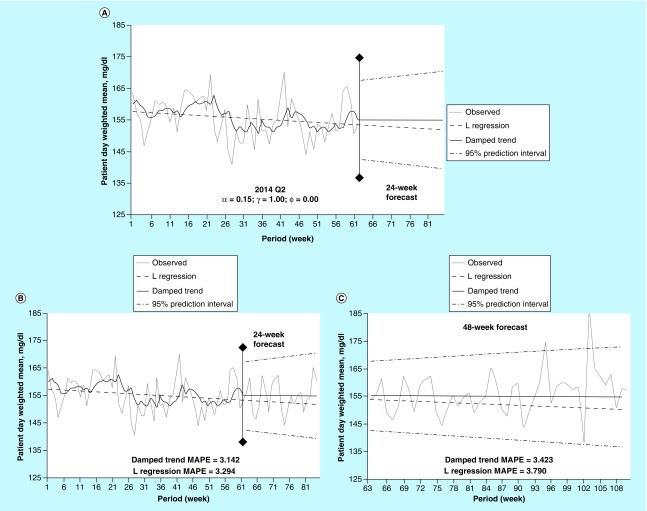
**Damped trend analysis for 2014.** **(A)** Damped trend and linear regression forecasts into the first 24 weeks without observed data for the time period. **(B)** The same comparisons as A with overlaying of observed values. **(C)** Damped trend and linear regression forecasts into the next 48 weeks of data. Each observation is the aggregation of a week-long period. MAPE: Mean absolute percent error; Q: Quarter.

The final example (Figure 4) illustrates the results of trending analyses for the second quarter of 2014 (Figure 4A). Similar to Example 2, a flat damped trend forecast contrasts with a decreasing-slope linear regression (Figures 4B & 4C). The damped trend α and γ coefficients, 0.15 and 1.00, respectively, suggest that a degree of smoothing and trending were required to optimize the model. This demonstrates the increased flexibility of the damped trend method, over a linear regression, to adapt to the behavior of the underlying data.

## Discussion

The damped trend method is considered the best practice within many industries [9]. However, to the authors’ knowledge, applying the model to forecasting inpatient glucose values represent a new and novel application of a 30-year-old method. The limitation of current glucometric analyses from a quality standpoint is that they rely on the use of retrospective data. Although helpful to direct quality improvement initiatives, standard glucometric methods still only provide a rear view mirror approach to glycemic control. Applying the damped trend forecasting method enables the switching of perspectives from what happened in the past to what may occur in the future. This provides an intriguing opportunity to become more proactive in the management of inpatient glucose management.

In order to test the utility of the damped trend methodology, we used 5 separate years of glucose data. Additionally, forecasts were made for the different years. In each instance, at least for this non-ICU data, when compared with a linear regression forecast, the projected glucose control agreed closely to what was actually observed during the first 2, 4, 6, 24 and 48 weeks of projections, indicating the good overall robustness of the model. The patient-day-weighted means in this study would be considered well within national guidelines for glycemic control and close to the national benchmark observed for the geographic region, hospital type and size [13,14].

In all scenarios, glucose forecasts were either flat or increasing/decreasing very gradually. This suggests that glucose data, despite week-to-week fluctuations, is generally consistent in the long term. For this reason alone, the comparison of the damped trend model to a linear regression was prudent. Why assume the burden of a more math-intense method if a simpler forecasting technique provides the same results? The authors contend that this analysis demonstrates the value of applying a more sophisticated method, despite the close agreement between the two models. In three of the four examples, applying forecasting via linear regression would have resulted in a decision to consider interventions to counter an upward or downward trend when, in fact, no such trend existed.

The analysis also demonstrated that both short- and long-term forecasts benefit from the additional sophistication of a damped trend method. In smaller patient populations where issues of hyperglycemia could be addressed quickly, shorter term forecasts might be more relevant than forecasts associated with patient populations spanning multiple geographic locations.

The damped trend exponential smoothing method, by design, dampens the effect of trends in the data. If the prediction heads in the direction of either hypo or hyperglycemia, then that is an indication of possible systemic issues driving glucose values in one direction or another. Not only can the method predict potential hypo/hyperglycemia in a patient population, it could be programmed to iteratively dive into subpopulations and shed light on the root cause of the trend.

There are some limitations to this analysis. The data are from a single institution, and the generalizability of the approach using data from other inpatient facilities should be evaluated. While every glucose measure cannot be regarded as independent, any autocorrelation that may exist in the data would not adversely impact the modeling of trends [7]. The 10–15% acceptance criteria for demand industries may not be relevant criteria for diabetes mellitus, but this criteria was the most relevant available. The results demonstrated here have lower percent errors, but further study is necessary to establish whether these smaller percentages are good enough. The greatest usefulness of damped trend analysis may be to assess the impact of an intervention geared to impact inpatient glycemic control processes. For instance, forecasting methods could be used to determine if the trends continue in the correct direction as an intervention progresses. Additionally, it should be understood that this is a population-based analysis. Data on unique patients are not considered, so conclusions regarding individual behavior cannot be made.

## Conclusion

To the authors’ knowledge, this report represents the first attempt to apply damped trend forecasting methodology to predict inpatient glycemic control data. This method addresses the previously identified limitation of managing glucose levels by retrospective analysis. Results suggest that the approach is suitable for both short- and long-range forecasting.

## Future perspective

Several areas of future work remain regarding the use of damped trend analysis of inpatient glucose data. For instance, applying the model to data derived from the ICU would be the next step. The model could also be tested by using data from individual specialties within the hospital (e.g., surgery or internal medicine), or other subpopulations within the hospital. Introducing conditions that might disturb the model and assessing the impact on the projected glycemic control is important. For instance, during 2015, a new POC-BG device was introduced into the authors’ hospital. Damped trend methodology could be used to determine how this technology changed forecasted glycemic control. Conducting trend analyses for other key glucometrics, such as hypoglycemia, would also be useful.

Additional analyses are required on how best to apply the technique as an adjunct to traditional quality improvement metrics, such as statistical process control. Comparison of damped trend analysis with other prediction methods would be of value. Finally, the model may also indicate when short-term forecasts are more accurate than longer term ones.

**Summary points**

- Analyses and reporting of glucose data are essential to quality improvement initiatives associated with the care of hospitalized patients with diabetes mellitus.
- The drawback of current approaches is that analyses rely on retrospective data-events that have already occurred.
- Ability to forecast inpatient glycemic control could provide an opportunity to anticipate unfavorable changes before they become a problem.
- Damped trend analysis – a forecasting technique used in industry – may have utility in predicting future inpatient glucose control.

**Methods**

- Point-of-care blood glucose data were extracted from laboratory information system from 2010 to 2014.
- Observed and calculated trends were determined over 62 weeks for different time segments from each year.
- Mean absolute percent error (MAPE) was used to calculate differences between observed and forecasted values.
- Results from two different forecasting methods (damped trend exponential smoothing and linear regression) were compared.

**Results**

- Damped trend forecast results for both short (2, 4 and 6-week) and long term (24 and 48-week) forecasts compared favorably to linear regression results, with 65% of scenarios demonstrating lower MAPE values for the damped trend method.
- When the damped trend method outperformed linear regression, the differences in error were most noticeable with the longer duration forecasts.
- In the four examples cited, the damped trend method depicted a visible attenuation of trending over time compared with linear regression. In three of the four examples, the attenuation was sufficient to delay, or render unnecessary and potential interventions.
- MAPE values for both forecasting techniques remained below the 10% accepted by demand industries.

**Discussion/conclusion**

- The greatest usefulness of damped trend analysis may be to better assess the impact of an intervention geared to impact inpatient glycemic control processes.
- For instance, forecasting methods could be used to determine if the trends continue in the correct direction as an intervention progresses.
- This report represents the first attempt to apply damped trend forecasting methodology to predict inpatient glycemic control data.

## References

[1] Goldberg PA, Bozzo JE, Thomas PG (2006). ‘Glucometrics’: assessing the quality of inpatient glucose management. *Diabetes Technol. Ther.*.

[2] Society of Hospital Medicine (2016). Overview: glycemic control implementation toolkit. http://www.hospitalmedicine.org/Web/Quality_Innovation/Implementation_Toolkits/Glycemic_Control/Web/Quality___Innovation/Implementation_Toolkit/Glycemic/Overview.aspx.

[3] American Association of Clinical Endocrinologists (2016). http://www.aace.com/.

[4] Saulnier GE, Castro JC, Cook CB (2014). Statistical transformation and the interpretation of inpatient glucose control data from the intensive care unit. *J. Diabetes Sci. Technol.*.

[5] Saulnier GE, Castro JC, Cook CB (2014). Statistical transformation and the interpretation of inpatient glucose control data. *Endocr. Pract.*.

[6] McKenzie E, Gardner ES (2010). Damped trend exponential smoothing: a modelling viewpoint. *Int. J. Forecast.*.

[7] Gardner ES, Mckenzie E (1985). Forecasting trends in time series. *Manage Sci.*.

[8] Bowerman BL, O'Connell RT, Koehler AB (2005). *Forecasting, Time Series, And Regression: An Applied Approach (4th Edition)*.

[9] Fildes R, Nikolopoulos K, Crone SF, Syntetos AA (2008). Forecasting and operational research: a review. *J. Oper. Res. Soc.*.

[10] Makridakis S, Hibon M (2000). The M3-competition: results, conclusions and implications. *Int. J. Forecast.*.

[11] Siegal AF (1990). *Practical Business Statistics With Statpad*.

[12] Gapenski LC (1993). *Understanding Health Care Financial Management: Text, Cases, And Models*.

[13] American Diabetes Association (2016). Diabetes care in the hospital. *Diabetes Care*.

[14] Bersoux S, Cook CB, Kongable GL, Shu J, Zito DR (2014). Benchmarking glycemic control in US hospitals. *Endocr. Pract.*.

